# A non‐invasive measure of bone growth in mammals: Validating urinary CTX‐I as a bone resorption marker through long‐bone growth velocity in bonobos

**DOI:** 10.1002/ece3.70326

**Published:** 2024-09-23

**Authors:** Verena Behringer, Ruth Sonnweber, Gottfried Hohmann, Jeroen M. G. Stevens, Jonas Verspeek, Tracy L. Kivell

**Affiliations:** ^1^ Endocrinology Laboratory, German Primate Center Leibniz Institute for Primate Research Göttingen Gottingen Germany; ^2^ Department of Behavioral and Cognitive Biology, Faculty of Life Sciences University of Vienna Vienna Austria; ^3^ Max Planck Institute for Evolutionary Anthropology Leipzig Germany; ^4^ Max Planck Institute of Animal Behavior Constance Germany; ^5^ SALTO Agro‐ and Biotechnology Odisee University of Applied Sciences Sint‐Niklaas Belgium; ^6^ Antwerp Zoo, Centre for Research and Conservation Royal Zoological Society of Antwerp Antwerp Belgium; ^7^ Department of Human Origins Max Planck Institute for Evolutionary Anthropology Leipzig Germany

**Keywords:** ape, biochemical bone marker, day‐to‐day variation, diurnal pattern, urine, validation study

## Abstract

Assessing bone growth trajectories in mammals is crucial for understanding life history dynamics, but the quantification of bone growth in natural settings can be challenging. Bone resorption markers that can be measured in urine, such as C‐telopeptide of type I collagen (CTX‐I), offer a non‐invasive solution to assess bone growth. Although measurement of urinary CTX‐I levels has been applied extensively in human studies, its use in other species is so far limited to a few clinical studies. To validate urinary CTX‐I as a bone resorption marker under less controlled conditions, we investigated within‐individual day‐to‐day variation, diurnal patterns, and sex and age‐specific variation in zoo‐housed bonobos (*Pan paniscus*). We then also correlated urinary CTX‐I levels with forearm growth velocity measures. We found a day‐to‐day variability in urinary CTX‐I levels of around 25%, comparable to human variation. Diurnally, CTX‐I levels decreased, aligning with observations in humans and other species. Both sexes showed an age‐related decline in urinary CTX‐I levels, with a steady decrease after the age of 10 years. Additionally, we found a positive correlation between forearm growth velocity and urinary CTX‐I levels across age in female, but not in male, bonobos. Our results demonstrate that urinary CTX‐I levels are a meaningful measure of bone growth and highlight its potential to examine bone growth trajectories also in wild populations to investigate life history dynamics.

## Graphical abstract

Our paper presents a novel approach in validating urinary CTX‐I levels as an indicator of bone growth, highlighting its potential as a valuable tool for assessing bone metabolism and growth dynamics in zoo‐housed and wild non‐human mammals. Consequently, our study contributes to advancing our understanding of skeletal physiology across species.

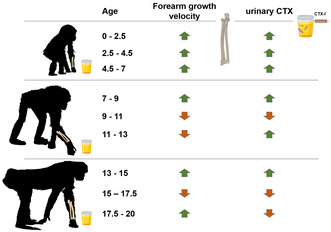

## 1 INTRODUCTION

The life history theory framework explores variations and interactions among maintenance, reproduction, and growth among individuals. Growth patterns, a classical life history trait (Stearns, 1992), encompass weight trajectories, muscle development, and bone growth. Quantifying growth in non‐human mammals, particularly in natural conditions, presents logistical and ethical challenges. Logistically, measuring growth in wild animals often requires repeated capturing and handling individuals to obtain accurate longitudinal data, which can only be done with specialized equipment (e.g., darting with anesthetic drug) and trained personnel. Ethically, capturing and handling induces stress and potential harm to the animals, which in turn can disrupt their natural behaviors, social structures, and overall well‐being. Also, some individuals of a population are more likely to be re‐captured than others, which may bias results to specific morphological and behavioral phenotypes (Webster & Rutz, 2020). These challenges necessitate the development of non‐invasive or minimally invasive methods for growth quantification in wild animals.

In humans, bone or somatic growth can be measured using various methods, such as biometric measures, radiography, or bone turnover markers (BTM) (Binkley et al., 2008; Szulc, 2018). BTM are widely used in clinical research (Szulc, 2018) and can be measured non‐invasively in urine, yet they are rarely applied in non‐human mammals. Here, we explore the utility of urinary BTM, specifically C‐telopeptide of type I collagen (CTX‐I), in assessing skeletal growth in zoo‐housed bonobos and potentially wild populations.

Bone is a metabolically active tissue, undergoing continuous modeling (growth) and remodeling (maintenance) throughout life (Currey, 2002). Osteoclasts, mediating bone resorption, are counteracted by osteoblasts, promoting bone formation (Jürimäe et al., 2009; van der Sluis et al., 2002). Activated osteoblasts and osteoclasts release enzymes, matrix proteins, and/or degradation products of the bone matrix into the circulatory system and/or urine. Therefore, evaluation of these products in serum or urine can reflect the ongoing interplay between skeletal modeling and remodeling (de Ridder & Delemarre‐van de Waal, 1998; van Coeverden et al., 2002).

CTX‐I is a commonly used marker for measuring bone resorption in humans (de Ridder & Delemarre‐van de Waal, 1998; Herrmann & Seibel, 2008; van der Sluis et al., 2002) because more than 90% of the organic matrix of bone comprises type I collagen (Vasikaran, 2008). Collagen crosslinks, like CTX‐I, are degradation products of this collagen (Herrmann & Seibel, 2008; Rosen et al., 2000). CTX is released into circulation in proportion to collagen degradation by osteoclasts (Urlacher et al., 2022). Therefore, CTX measurement reflects changes in bone metabolism (Herrmann & Seibel, 2008). In humans, CTX‐I is small enough to be metabolized and cleared by the kidneys and thus can be measured in urine (Herrmann & Seibel, 2008; Komi et al., 2004). Urinary CTX‐I levels correlate with serum CTX‐I levels (rho = 0.87, Table 2 in Herrmann & Seibel, 2008; Szulc et al., 2017). Therefore, urinary excretion rate of crosslink‐containing collagen fragments serves as an index of bone collagen degradation (Mora et al., 1998), providing a non‐invasive way to assess bone resorption in humans.

Nevertheless, clinical studies in humans show that the measurement and interpretation of urinary CTX‐I requires careful consideration for several issues. Studies have found substantial within‐subject variability in BTM (de Ridder & Delemarre‐van de Waal, 1998; Herrmann & Seibel, 2008). CTX‐I levels show a within individual day‐to‐day variation of more than 20% (Hannon & Eastell, 2000). Moreover, urinary and serum CTX‐I levels are subjected to diurnal variation, with the highest levels in the morning and lowest levels in the afternoon, displaying daily amplitudes of 20%–30% (Aoshima et al., 1998; reviewed in Tian & Ming, 2022). Furthermore, urine and serum CTX‐I levels show ontogenetic‐related variation. During the first postnatal month of life levels increase, followed by a decline until the age of three, although levels remain higher than those of adults, and the pubertal growth spurt is associated with an increase in CTX‐I levels (Herrmann & Seibel, 2008; Szulc et al., 2000), with urinary CTX‐I levels being highest during early puberty in girls and boys (reviewed in Yang & Grey, 2006). This increase in CTX‐I levels during puberty aligns with periods of considerable bone growth, mineralization, and increased bone turnover (van Coeverden et al., 2002; van der Sluis et al., 2002). Thus, CTX‐I levels reflect linear growth rates throughout childhood and during adolescent growth spurts (Rauchenzauner et al., 2007; Szulc et al., 2000), demonstrating the utility of CTX‐I as a biomarker of bone growth (Urlacher et al., 2022).

Measuring growth patterns in natural conditions in non‐human mammals can be challenging due to ethical and health regulations (e.g., no physical contact, limited or no medical interventions) and logistical challenges of the environment. Investigation of CTX‐I and growth in non‐human mammals is limited. Urinary CTX‐I levels have been investigated in a clinical context in dogs (Ahner et al., 2019), cats (DeLaurier et al., 2004) and in rats (Ishikawa et al., 2004). Studies in captive non‐human primates have found that bone resorption marker (BRM) in urine and serum decline after suppression of bone turnover rate (Angeliewa et al., 2004), and BTM were higher in younger compared to mature long‐tailed macaques (*Macaca fascicularis*, Legrand et al., 2003). In wild non‐human primates, non‐invasive techniques such as photogrammetry are sometimes used to estimate physical (somatic) size growth trajectories (e.g., *Macaca assamensis*, Anzà et al., 2022; *Papio cynocephalus*, Levy et al., 2023; *Pongo* spp., Schuppli et al., 2016); however, the environment (e.g., dense foliage) can restrict photogrammetry. To our knowledge, Sandel et al. (2023) is the only study to date to measure BTM in a wild primate. They measured urinary levels of osteocalcin (bone deposition) and NTX (bone resorption) in wild chimpanzees (*Pan troglodytes*) and found peaks in BTM in males between ages of 9 and 11 years (Sandel et al., 2023). This showed that measures of BTM from urine may offer a promising, but largely unexplored, avenue to quantify skeletal growth non‐invasively in (wild) non‐human mammals.

In our study, we tested the correlation between urinary CTX‐I levels, as a BRM, and measures of bone growth velocity (i.e., change in forearm length) in zoo‐housed bonobos (*Pan paniscus*) to validate the use of urinary CTX‐I as a robust measure of bone growth. We analyzed urinary CTX‐I because it is regarded as the preferred BRM by the International Osteoporosis Foundation and the International Federation of Clinical Chemistry and Laboratory Medicine (Vasikaran et al., 2011). To establish urinary CTX‐I measurements as a biologically meaningful BRM, we took a three‐step (validation) approach, expecting to find comparable patterns of CTX‐I secretion as has been previously described in humans given the close evolutionary relationship between humans and bonobos (Prüfer et al., 2012). First, we assessed inter‐individual day‐to‐day variability of urinary CTX‐I levels in bonobos. We predicted large variation in daily urinary CTX‐I levels as has been documented in humans (Ju et al., 1997). Second, we investigated diurnal patterns in urinary CTX‐I levels. We predicted a decline in CTX‐I levels throughout the day, consistent with previous diurnal patterns found in humans and other mammals (Tian & Ming, 2022). Third, we correlated urinary CTX‐I levels with forearm growth velocity (FGV) (Behringer et al., 2016; Berghaenel et al., 2023) to test two additional predictions: (3a) If urinary CTX‐I is a useful measure of bone growth in bonobos, as suggested by human studies (Rauchenzauner et al., 2007; Szulc et al., 2000), we expected elevated CTX‐I levels during periods of increased long‐bone growth rates. Specifically, we predicted that younger individuals to have higher urinary CTX‐I levels than older individuals. (3b) Bonobos, like humans, express sex differences in the adolescent growth spurt (Berghaenel et al., 2023). If these sex differences are reflected in urinary CTX‐I levels, we predicted an earlier decline in CTX‐I levels in females compared with males.

## 2 MATERIALS AND METHODS

### 2.1 Subjects

We collected a total of 239 urinary samples on a total of 52 bonobos (26 females and 26 males) across all ages, housed in 11 European and North American Zoos. Sample collection method involved direct sampling on plastic sheets or retrieval from the floor using disposable plastic pipettes, with subsequent transfer into plastic vials. The frozen samples were transported and analyzed at the German Primate Center, Göttingen, Germany.

To account for variations in urine concentration based on individuals' hydration status, specific gravity (SG) was measured in all samples using a digital hand refractometer (Miller et al., 2004). We chose to use SG due to concerns that day‐to‐day variation in CTX‐I levels may be exacerbated when correcting with creatinine (Legrand et al., 2003). Additionally, creatinine levels are influenced by factors such as sex, age, activity, and diet, as it is a by‐product of muscle activity (Miller et al., 2004). Given that our dataset included both sexes and a wide age range, SG is the more suitable method for correcting urine concentration. We discarded samples with an SG <1.003 (Sabbi et al., 2020). We used different subsets of the data for the analyses, as described below.

### 2.2 Urine for day‐to‐day variation

Day‐to‐day variation in urinary CTX‐I levels was assessed using samples from four zoo‐housed female bonobos, none of whom were pregnant or lactating. In total, 13 samples were collected within 2–3 weeks per female (urine collection period: female1 = May 01–13, 1992; female2 = September 15–29, 1995; female3 = March 01–20, 2015; female4 = June 01–22, 1994). All samples were collected around the same time of day in each female.

### 2.3 Long‐term datasets

To investigate diurnal urinary CTX‐I secretion patterns in relation to age, sex, and growth velocity in bonobos we used two long‐term datasets on individuals between the ages of 0 and 20 years (beyond that age, bonobos are not expected to grow anymore, Berghaenel et al., 2023). The first dataset included urinary CTX‐I levels measured to assess daily variability and its correlation with age and sex. The second large dataset on forearm growth measurements was used to quantify individual bone growth velocity trajectories, which was then correlated with age‐related urinary CTX‐I level excretion patterns. We outline both sets of analyses in more detail below.

#### 2.3.1 Long‐term urinary data

After excluding all individuals older than 20 years of age, the urine dataset comprised a total of 187 urine samples of 48 individuals (mean = 4 +/− 4.2, median = 1, range = 1–18 samples per individual). Of those, 22 individuals were females and 26 were males. The chronological age was known for all individuals in the dataset, and ages ranged from less than 1‐month‐old to 19.3 years, with females averaging 7.2 years and males averaging 8.0 years at the time of sample collection. Urine samples were collected between September 1998 and March 2022. Collection occurred throughout the day, between 07:00 and 18:00 h. Samples were stored at around −18 – −20°C. Long‐term stability studies on CTX‐I and especially on urinary CTX‐I are scarce, but bone resorption markers, including CTX‐I, are stable when frozen (reviewed in Szulc et al., 2017). For example, plasma and serum CTX‐I were unaffected by storage at −20°C up to 3 years (Qvist et al., 2004).

#### 2.3.2 Forearm growth data

The forearm dataset included a total of 364 forearm measurements (see below). For a total of 81 individuals (47 females, mean = 4.4 +/− 2.62, median = 4, range = 1–10; 34 males, mean = 4.68 +/− 2.85, median = 4, range = 1–10, age ranged from 3 months to 20 years) we had between one and ten forearm length measurements (mean = 4.5 +/− 2.7, median = 4). Forearm measurements from bonobos were collected using a transparent Plexiglas tube (125 × 1400 mm, with a metric scale on each side) connected to the enclosure. This method was previously described and validated for the use in bonobos (Behringer et al., 2016). The process involved capturing digitized images from video recordings (Sony HDR–CX115EB Full HD Camcorder) as individuals extended their arms into the plastic tube for a reward. Image analyses were performed in ImageJ (Abràmoff et al., 2004).

### 2.4 Urinary CTX‐I measurement

A total of 239 urine samples (from the day‐to‐day and the long‐term datasets) were processed at the Endocrinology Laboratory of the German Primate Center, Göttingen, Germany. For CTX‐I measurement, we used the commercial sandwich assay (two highly specific antibodies) Urine BETA CrossLaps®(CTX‐I) ELISA (REF AC‐05F1) from Immunodiagnostic Systems (IDS). The assay is designed for quantifying degradation products of C‐terminal telopeptides of Type I collagen in human urine. All samples were run in duplicates following instructions from the IDS company (Data S1). Our results are expressed in ng/ml corrected for specific gravity (SG).

To assess urine sample dilution, we conducted a parallel dilution along the standard curve of the ELISA. Six samples were diluted four times (pure, 1:2, 1:4, and 1:8) following the manual, using CrossLaps Standard 0. Dilution showed displacement to the standard curve. Measurements were found to be optimal with pure samples when SG was between 1.003 and 1.005, and 1:2 dilutions when SG was higher than 1.005.

### 2.5 Datasets and data preparations for statistical analyses

To prepare our data for statistical analyses we performed the following steps.

#### 2.5.1 Long‐term urinary CTX‐I‐dataset

We transformed the time of sample collection from an hour and minute format to minutes.

#### 2.5.2 Forearm‐dataset

To calculate forearm growth velocity (FGV), we subtracted the former of two subsequent forearm length measurements from the latter measurement and divided the difference of forearm length measurements by the number of days that passed between the two measurements. Since forearm growth takes place in between two subsequent measurements we assigned the date in between two measurements as the “velocity date.” Then we subtracted the birthdate of an individual from its velocity dates to get the age of the individual (in days) at the time of that estimated FGV.

To compare FGV and urinary CTX‐I level patterns in a common standardized scale, we mean centered and standardized (to two standard deviations) the age gradient across both datasets (Gelman, 2008), and mean centered and standardized to two standard deviations the sex (both datasets) as well as the time (CTX‐I‐dataset) variable. This procedure has several advantages: (i) it enhances the interpretability of the results as changes in the response variable will be associated with a change of two standard deviations in the predictor variable, (ii) it is easier to compare the relative effects of predictors (that often differ in scale and measurement units) on the response variables, and (iii) the optimization process of model fitting and the setting of reasonable priors is improved. Lastly, we forced all velocity measurements to be positive values (93 velocity measurements were negative, ranging between −0.007 cm/day and −0.000005 cm/day, which is caused by small errors in measuring forearm length) and then log‐transformed the response variables urinary CTX‐I level and FGV.

### 2.6 Statistical analyses

To investigate diurnal pattern, age, and sex variability (CTX‐I model) and the correlation between variation in urinary CTX‐I levels and growth velocity (comparing CTX‐I with FGV model), we employed Bayesian regression models using the brm() function from the *brms* package in R. Prior to model fitting, we assessed the distributions of both log‐transformed FGV and log‐transformed urinary CTX‐I levels by plotting histograms. These plots revealed that both variables exhibited a left‐skewed distribution. To accommodate the observed asymmetry in the response variables, we selected the skew‐normal probability distribution for modeling with mu (mean or location), sigma (standard deviation or scale parameter), and alpha (skewness parameter) as identity functions (see Data S1). These parameters served as key links within our models, allowing us to consider the underlying probability distribution of the response variables and the relationships between these parameters and predictors. Our models ran five Markov chains in parallel, which assessed convergence and generated robust parameter estimates. Each chain underwent 10,000 iterations without thinning, with the initial 2000 iterations in each chain allocated for adaptation and tuning. Parallel processing was performed using two CPU cores. To ensure efficient exploration of the posterior distribution during the sampling process, we had set the adapt‐delta parameter to 0.99. The models were sampled using the NUTS (No‐U‐Turn Sampler) algorithm. Effective sample sizes and potential scale reduction factors are reported in the model outputs.

The velocity model included the log‐transformed FGV as the response variable and the interaction between sex and age of the individual as predictor variables to model sex‐specific growth velocity patterns across age (Berghaenel et al., 2023). Since we employed a repeated measures design (Wallace & Green, 2013), we added a random intercept for the individual, a random slope of age for the individual, and a random intercept for the zoo where an individual was housed to account for potential individual‐ and site‐specific variation in growth patterns (Snijders & Bosker, 2012). Both, FGV and CTX‐I levels across ages are expected to follow a non‐linear pattern (e.g., Berghaenel et al., 2023; Mora et al., 1998). Therefore, we used a smooth function of age (basis splines) to model the curves of the two variables of interest (Meyer, 2005). In the context of our study, splines are used to create smooth curves that represent the age‐related changes in FGV and urinary CTX‐I levels. Specifying smooth function of age with ten knots allowed us to model the FGV curves of the two sexes in detail to capture age‐related fluctuations (growth spurts) while avoiding overfitting (see also Data S1). Furthermore, to constrain model estimates and to provide information about the expected patterns of the parameters, we set priors (see Data S1) in the model specification (McElreath, 2018). Since female and male bonobos differ in their forearm growth pattern (Berghaenel et al., 2023), we had set informative priors (specifying small standard deviations) for expected sex differences in FGV patterns between the ages of five and eleven (females having an earlier, shorter growth spurt than males, Berghaenel et al., 2023).

To ensure the quality of our model, we conducted a comprehensive set of model diagnostics and compared our model to alternative ones (smooth function of age with six and eight knots; different priors). The fit of competing models was assessed by (i) comparing predictive performance (using the *loo* package, Vehtari et al., 2017, 2023), (ii) examining parameter estimates and credible intervals, and (iii) plotting predictive checks. To validate the convergence of the model, we utilized the Gelman‐Rubin diagnostics (Gelman & Rubin, 1992). Additionally, we visually examined posterior distributions for all model variables, posterior predictive check plots, residual plots, observed versus predicted plots, and assessed autocorrelation. To evaluate the robustness of our results to prior specifications, we conducted sensitivity analyses by fitting alternative models with varying levels of prior strength and different probability distributions, utilizing the *loo* package (Vehtari et al., 2017).

As the goal of our study was to test whether urinary CTX‐I levels follow a pattern parallel to the FGV pattern, we fitted a CTX‐model following the same parameter settings as the growth velocity model. Thus, the CTX‐model included the log‐transformed urinary CTX‐I levels as a response variable and the same fixed and random effects as the velocity models. Additionally, the time of sample collection (mean centered and standardized to two standard deviations) was included in CTX‐models to account for circadian variation in CTX‐I levels (Aoshima et al., 1998; Mora et al., 1998). We used the same priors (Data S1), informing about the expected sex‐specific patterns across age (Mora et al., 1998), but specified them to be less informative than in the velocity‐model to maintain flexibility in the model calculations and to achieve data‐driven results.

From both the velocity‐ and the CTX‐model, we extracted estimates and confidence intervals of the population‐level effects to examine sex‐specific patterns of FGV and urinary CTX‐I levels across ontogeny. Estimates and confidence intervals for males were derived by adding the estimates and confidence intervals for the sex differences to the estimates and confidence intervals of the main effects of each age spline of the age smooth curve. To test whether FGV and urinary CTX‐I levels across ages follow similar patterns within each sex, we ran Spearman correlation tests by sex. We correlated estimates of FGV and urinary CTX‐I levels for each of the nine age splines of the age smooth function.

## 3 RESULTS

### 3.1 Day‐to‐day variation

Day‐to‐day variation of urinary CTX‐I levels for each of the four females is shown in Table 1. Mean day‐to‐day variability (CV) in urinary CTX‐I levels across all females was 24.7%.

**TABLE 1:** Day‐to‐day variability (CV) of urinary CTX‐I levels in four adult females.

ID	*N*	Mean	Median	Std. dev.	Std. error	Min.	Max.	CV (%)
Female1	13	3.3	3.06	0.96	0.28	1.86	5.2	29.1
Female2	12	3.75	3.90	0.64	0.18	2.44	4.86	17.1
Female3	11	4.69	4.42	1.49	0.45	3.03	7.06	31.8
Female4	12	1.14	1.10	0.24	0.07	0.87	1.73	21.1

*Note*: In four samples the measure of urine concentration, specific gravity, was less than 1.003, and samples were excluded.

Abbreviations: ID, Individual; Max, Maximum; Min, Minimum; *N*, Number of urine samples; Std.dev, Standard deviation; Std.error, Standard error.

### 3.2 Diurnal variation

**FIGURE 1:**
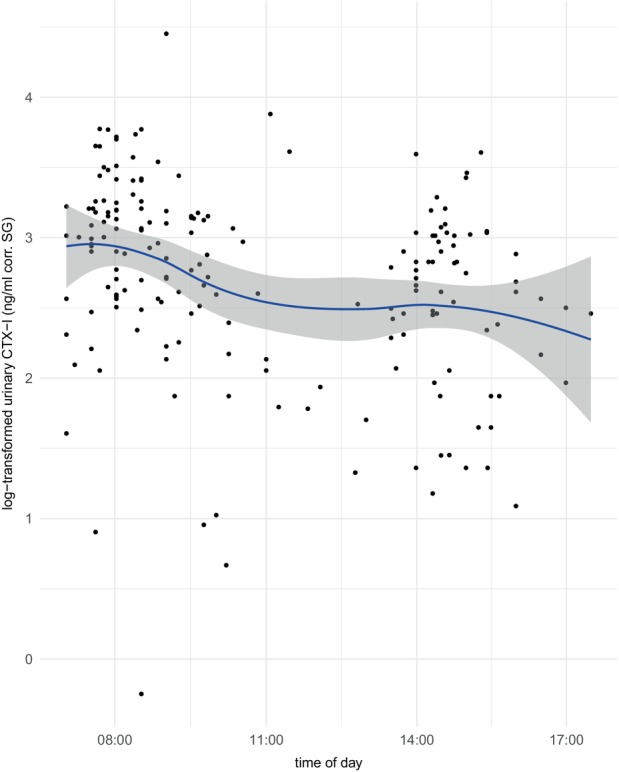
Diurnal pattern of urinary CTX‐I levels. Urinary CTX‐I levels (on the y‐axis) decreased throughout the day (time of day on the x‐axis). The blue line represents the regression line, the gray area represents 95% confidence intervals, and black dots are 187 urinary samples of 48 bonobos.

To assess a potential diurnal pattern in urinary CTX‐I levels in bonobos, we included the time of sample collection as a predictor variable in our CTX‐model. In the CTX‐model, the population‐level effects for the time of day variable show a negative estimate of −0.23 (SE = 0.11, lCI = −0.44, uCI = −0.02), suggesting throughout the day, urinary CTX‐I levels decrease (Figure 1).

### 3.3 CTX‐I levels in relation to FGV

Examining residuals, observed versus predicted plots, posterior distributions, and posterior predictive checks from our velocity‐ and CTX‐model indicated that model assumptions were met for both models. The models converged well as suggested by Gelman Rubin diagnostics (Gelman & Rubin, 1992), and we did not find indications for issues with autocorrelation in the data. Information on model checks and assumptions can be found in Data S1.

**FIGURE 2:**
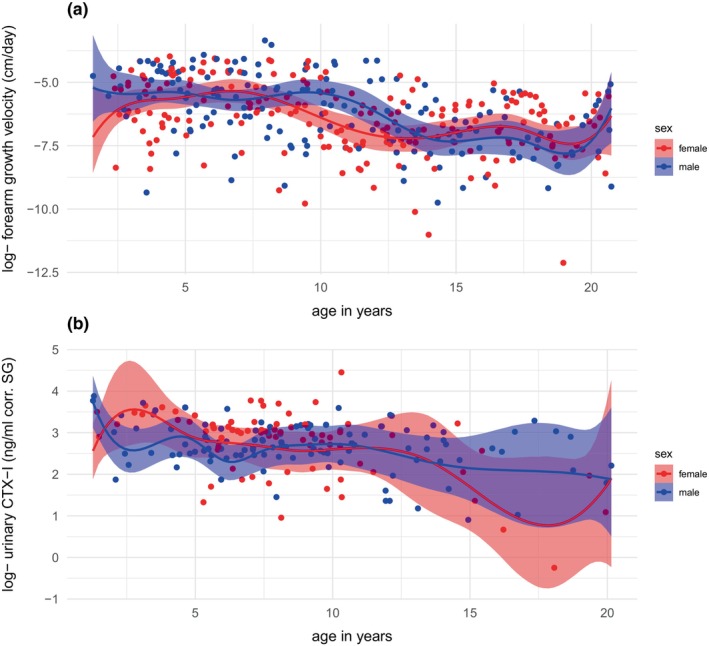
Patterns of forearm growth velocity (log‐transformed) (a) and urinary CTX‐I levels (log‐transformed ng/ml corrected for specific gravity) (b) across age (in years) for each sex. The plots represent the estimated population values of forearm growth velocity and urinary CTX‐I levels across the smooth function of age (solid lines) with 95%‐confidence intervals and individual data points (individual dots).

Model results (Table 2) showed that females have a strong increase in FGV in the first four age splines (approximately ages 0 – 7 years, Figure 2a, in red). FGV decreased thereafter and reached nadir in the sixth age spline (at around 13 years of age). Female urinary CTX‐I levels (Figure 2b, in red) showed a comparable pattern, although here a strong increase was found only in the first age spline (approximately 0 – 2.5 years). Compared to females, males had high FGV (Figure 2a, in blue) from the first measurements onwards and showed only minor fluctuations in FGV throughout the first five age splines (up to ~11 years of age). In males, FGV started to decrease in the sixth age spline, at around 11 years of age. Male urinary CTX‐I levels increased after birth and peaked in the second age spline (at around 4 years of age, Figure 2b, in blue) and showed a slow downward trend thereafter. It is important to note that all of these described patterns are associated with large credible intervals that include zero (Table 2). These intervals indicate a wide range within which the true population level parameter value is expected to be, suggesting a potentially large inter‐individual variation in age‐related growth patterns.

**TABLE 2:** Results of the effect of chronological age on forearm growth velocity and urinary CTX‐I levels by sex.

Age spline: approximate age range	Females		Change	Males		Change
	Forearm growth velocity	Urinary CTX‐I		Forearm growth velocity	Urinary CTX‐I	
	*β* (lCI–uCI)	*β* (lCI–uCI)		*β* (lCI–uCI)	*β* (lCI–uCI)	
1: 0–2.5	1.52 (−1.38–3.91)	1.85 (−0.29–4.36)	↑↑	−0.58 (−5.27–3.68)	−2.14 (−5.94–1.56)	↓↓
2: 2.5–4.5	1.18 (−0.70–2.78)	0.27 (−0.97–1.50)	↑↑	0.06 (−3.25–3.11)	0.03 (−1.73–1.27)	↑↑
3: 4.5–7	2.02 (−0.20–3.75)	0.21 (−0.62–1.05)	↑↑	−0.88 (−5.18–1.24)	−1.74 (−2.90 – −0.97)	↓↓
4: 7–9	1.03 (−0.99–2.62)	0.11 (−0.75–0.92)	↑↑	−0.04 (−2.43–1.94)	−1.04 (−1.70 – −0.35)	↓↓
5: 9–11	−0.15 (−2.32–1.64)	−0.05 (−0.89–0.78)	↓↓	−0.67 (−2.22–0.39)	−1.04 (−1.96 – −0.26)	↓↓
6: 11–13	−0.24 (−2.44–1.66)	0.10 (−1.04–1.25)	↓↑	−2.81 (−6.15–0.64)	−0.95 (−2.75–0.26)	↓↓
7: 13–15	0.96 (−1.36–2.97)	0.08 (−3.19–3.19)	↑↑	−1.32 (−6.55–1.64)	−1.80 (−4.70–1.10)	↓↓
8: 15–17.5	−1.20 (−3.67–1.02)	−3.09 (−7.29–1.26)	↓↓	−3.70 (−8.65–1.06)	−1.63 (−5.13–0.18)	↓↓
9: 17.5–20	0.76 (−1.72–3.02)	−0.64 (−2.87–1.73)	↑↓	−0.90 (−4.54–2.51)	−1.81 (−4.04–0.42)	↓↓

*Note*: Estimates (*β*), lower 95% confidence intervals (lCI), and upper 95% confidence intervals (uCI) are derived from the velocity‐ and CTX‐model, respectively. Estimates and confidence intervals for males are derived by adding the estimates and confidence intervals for the sex differences to the estimates and confidence intervals of the main effects of each age spline of the smooth curve of age.

Females and males differed from each other in regard to dynamic changes across age in both FGV and urinary CTX‐I levels (Table 3). In particular, females had higher FGV as well as urinary CTX‐I levels in the first age spline (between 0 and 2.5 years of age). Furthermore, we found sex differences in FGV in the third to the seventh age spline (approximately 5 – 13 years of age).

**TABLE 3:** Relative sex differences in forearm growth velocity and urinary CTX‐I levels across the first 20 years of life.

Age spline: Approximate age range	*β* (lCI–uCI) for forearm growth velocity	*β* (lCI–uCI) for urinary CTX‐I levels
1: 0–2.5	−2.10 (−3.89 – −0.23)	−3.99 (−6.65 – −1.70)
2: 2.5–4.5	−1.12 (−2.55–0.33)	−0.24 (−1.77–1.34)
3: 4.5–7	−2.90 (−3.29 – −2.51)	−1.95 (−2.72 – −1.18)
4: 7–9	−1.07 (−1.45 – −0.68)	−1.15 (−1.93 – −0.35)
5: 9–11	−0.52 (−0.90 – −0.13)	−0.99 (−1.73 – −0.23)
6: 11–13	−2.57 (−4.13 – −1.02)	−1.05 (−2.45–0.32)
7: 13–15	−2.28 (−4.27 – −0.33)	−1.88 (−5.25–1.67)
8: 15–17.5	−2.50 (−4.98–0.04)	1.46 (−3.81–6.31)
9: 17.5–20	−1.66 (−3.85–0.49)	−1.17 (−3.87–1.61)

*Note*: The values indicate the estimates for the differences in forearm growth velocity and urinary CTX‐I levels for each age spline, with males being the reference category.

**FIGURE 3:**
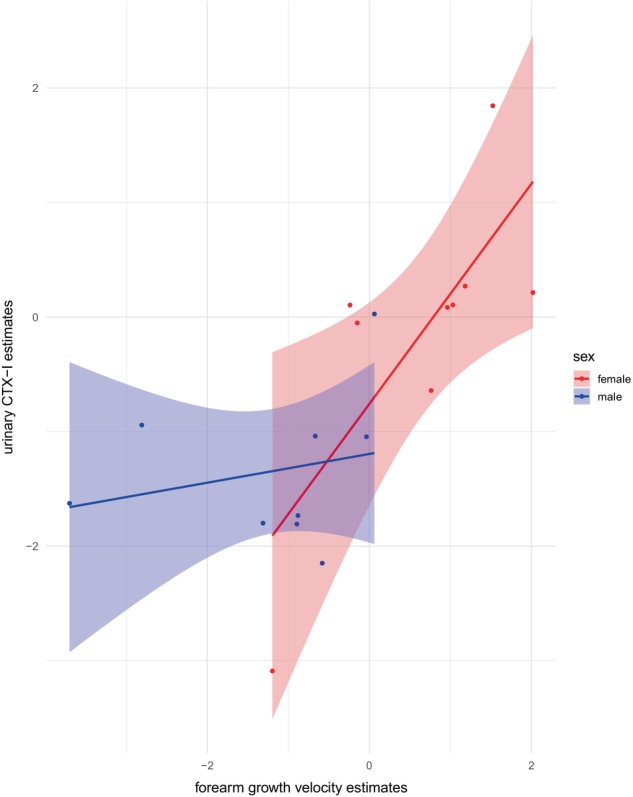
Correlation between estimates for forearm growth velocity and urinary CTX‐I levels across age in females (red) and males (blue). Estimates of forearm growth velocity across the smooth function of age showed a positive correlation with estimates of urinary CTX‐I levels across ontogeny in females, but not in males. Dots present the estimates for the eight intervals.

We then extracted the fixed effects for the different age splines for each sex from both the velocity and the CTX‐model and merged these in one data frame. For females (Figure 3), there was a significant positive correlation between the estimates of FGV and the estimates of urinary CTX‐I levels for the individual splines of the smooth curve of age (*r*(7) = 0.83, *p* = .008; Figure 3). In contrast, in males there was no such correlation between estimates of FGV and urinary CTX‐I levels across the smooth function of age (*r*(7) = 0.18, *p* = .644; Figure 3).

**FIGURE 4:**
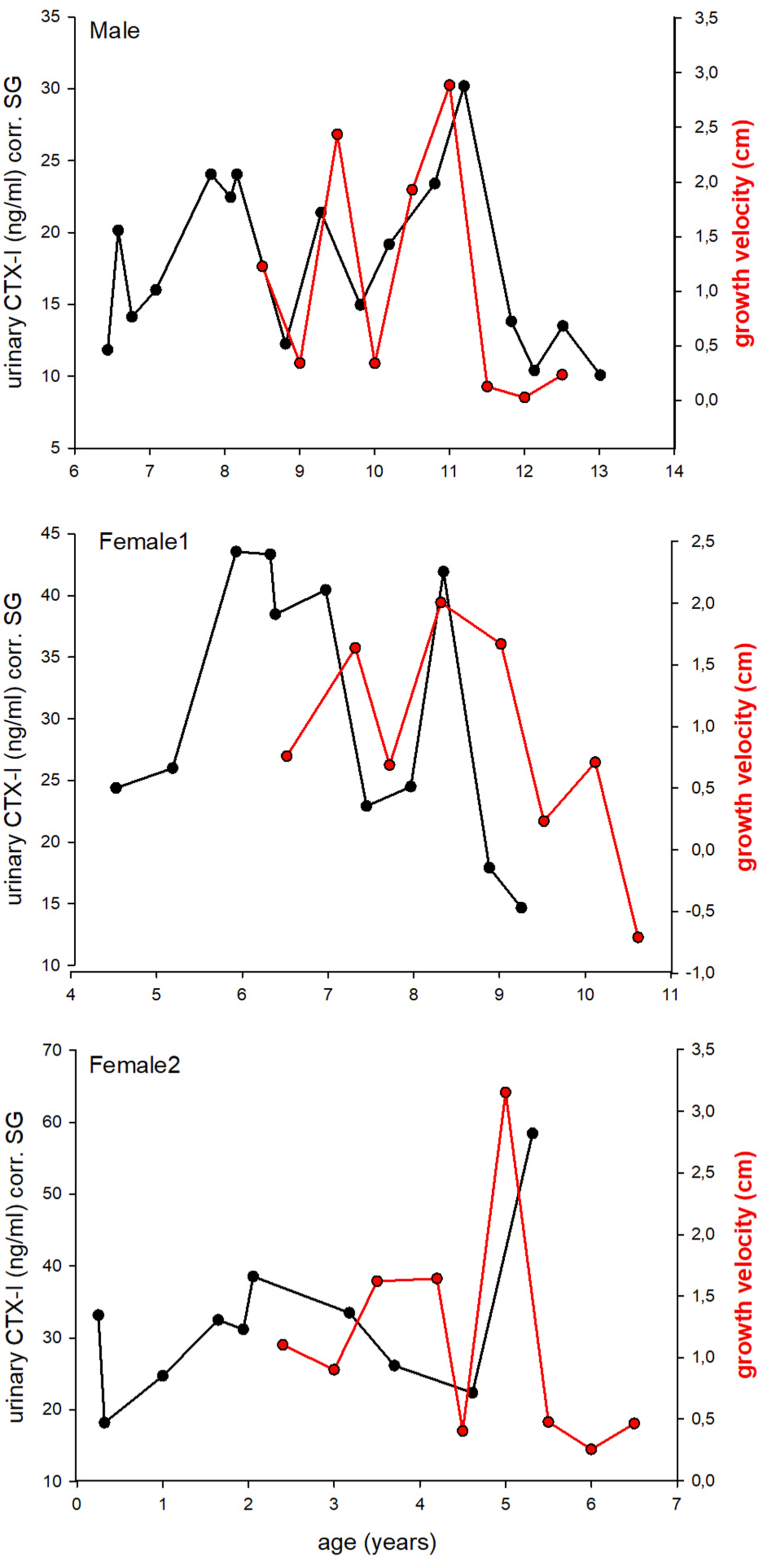
Association between forearm growth velocity (black) and urinary CTX‐I levels (red) within three bonobos. Axes are displayed on different scales. Black dots present a urinary CTX‐I measurement and red dots present a growth velocity calculation between two forearm measurements.

We visually compared growth velocity with urinary CTX‐I levels within three individuals (one male and two females), in which urine samples were always collected in the morning before bonobos were provided with food, to exclude nutritional and circadian effects on urinary CTX‐I levels (Figure 4). Individual profiles show a positive association between FGV and urinary CTX‐I levels in all three subjects.

## 4 DISCUSSION

This study investigated for the first time urinary CTX‐I measurements as a method to non‐invasively quantify bone growth in bonobos that could also be applied to other mammals, both in zoos and in the wild. Following previous results found in humans, we tested predictions about daily and age‐related variation in CTX‐I levels in female and male bonobos. Our findings supported our predictions: we found the expected large day‐to‐day variation and a diurnal decline in urinary CTX‐I levels throughout the day. We further tested the value of urinary CTX‐I as biomarker of bone growth in bonobos through the association of CTX‐I levels and forearm bone growth, finding a positive correlation in females but not in males. Each of these results are discussed in more detail below.

### 4.1 CTX‐I day‐to‐day variability and diurnal pattern

Day‐to‐day variability in female bonobo urinary CTX‐I levels was approximately 25%, nearly identical with previous results in humans (mean day‐to‐day variations of 23%, Ju et al., 1997). The variation of average urinary CTX‐I across the four females in our study (17%–32%) was also comparable to that found in humans (12%–35%, Ju et al., 1997).

In humans, several factors have been shown to contribute to this day‐to‐day variation in CTX‐I levels. First, urine concentration correction with creatinine levels may account for some of this variation (Legrand et al., 2003). Second, nutritional factors, such as the intake of calcium, vitamin D, protein, phosphate, glucose, fat, or a generally a meal can reduce serum CTX‐I levels by up to 50% within 60–120 min after consumption (Herrmann & Seibel, 2008; reviewed in Szulc et al., 2017). While bonobo samples for this part of the study were collected at consistent times for each individual, the potential influence of varying food intake on CTX‐I levels remains uncertain for the bonobos. Third, using urine samples of adult cycling females introduces another layer of variability since in humans lower CTX‐I levels are found during the luteal phase of the menstrual cycle compared to the follicular phase, with serum CTX levels ranging from 9% to 14% in humans (e.g., Mozzanega et al., 2013; Szulc et al., 2017). As most urine samples (three of four females) in our study were part of a previous study (Heistermann et al., 1996), we know that those were collected during both the luteal and the follicular phases. Assuming the same bias found in humans also applies to bonobos, the menstrual cycle may account for some of the variation in bonobo CTX‐I levels. Finally, physical activity can also affect CTX‐I levels, such that intensive physical training in humans leads to a moderate increase in CTX‐I levels (Szulc et al., 2017). The extent of physical activity in our study females before urine collection is not known.

Urinary CTX‐I levels exhibited a significant 45% decline throughout the day, consistent with previous findings in humans and other species (Aoshima et al., 1998; Ju et al., 1997; Tian & Ming, 2022). For example, in humans, diurnal changes in urinary CTX levels were found to decline by 54% – 57% throughout the course of the day (Aoshima et al., 1998; Ju et al., 1997).

While these day‐to‐day variations as well as diurnal decline in CTX‐I levels contribute to overall CTX‐I variation, they also validate that we measured biologically meaningful CTX‐I levels in bonobo urine. Furthermore, these results highlight the need to standardize or control for influencing factors, such as food intake or exercise, in sampling designs (Martin et al., 2021; Szulc et al., 2017) or in statistical models. Finally, as the aforementioned factors introduce large variation (noise) in CTX‐I levels, the effect size of other predictors of interest must be fairly large in order to be able to successfully detect a relationship with urinary CTX‐I levels.

### 4.2 Age‐related changes in CTX‐I

In both sexes of bonobos, urinary CTX‐I levels decreased steadily after the age of 10 years, similar to age‐related changes found in serum and urinary CTX measurements in humans (Herrmann & Seibel, 2008), serum osteocalcin assessment in rhesus monkeys (*M. mulatta*, Cahoon et al., 1996), serum bone formation marker in long‐tailed macaque (*M. fascicularis*, Legrand et al., 2003), and urinary NTX and osteocalcin in chimpanzees (*P. troglodytes*, Sandel et al., 2023). Overall, we found large confidence intervals in bonobo urinary CTX‐I levels, possibly related to the sources of variation discussed above.

We expected to find highest urinary CTX‐I levels in adolescent bonobos, between the ages of 6 and 10 years (Berghaenel et al., 2023), because this is the period of the most rapid gain in bone mass in humans (Chevalley & Rizzoli, 2022). Both female and male bonobos in our study showed an increase in urinary CTX‐I levels during adolescence. This finding builds upon previous studies in bonobos (Berghaenel et al., 2023) and chimpanzees (Sandel et al., 2023), showing that at least these two ape species experience an adolescent growth spurt; a developmental phase that was previously thought to be unique to humans (Bogin, 2021). However, females showed highest levels of urinary CTX‐I around 4 years of age. This difference suggests potential sex‐specific investment in skeletal growth, possibly influenced by the onset of sexual maturation. Hormonal evidence indicates that female bonobos have an earlier onset of pubertal maturation (Behringer et al., 2014) and also an earlier onset of weight gain than males (Berghaenel et al., 2023).

### 4.3 Relation of FGV and CTX‐I levels

We found a positive correlation between population estimates of FGV and urinary CTX‐I levels in female bonobos but not males. Our results in female bonobos are similar to increases in urinary bone resorption markers during childhood and during the adolescence growth spurt in humans (Mora et al., 1998; Rauch et al., 2009). However, male bonobos showed a different urinary CTX‐I pattern compared to their growth velocity trajectory. This sex difference may be attributed to the broader role of CTX‐I in reflecting not only (forearm) long bone growth but also overall skeleton modeling and remodeling in general (de Ridder & Delemarre‐van de Waal, 1998; Jürimäe et al., 2009; Schönau & Rauch, 1997). Moreover, human studies show that CTX levels are associated with maturation status rather than chronological age (e.g., Fares et al., 2003), and thus our data may be capturing known differences in maturation life history among female and male bonobos (Berghaenel et al., 2023). Furthermore, within a population, there is considerable heterogeneity with respect to individual pubertal stages (Marowska et al., 1996). Given that our model compared population growth velocity estimates with population CTX‐I levels, it is possible that individuals within the population vary within the large confidence intervals. Our within‐individual comparison of growth velocity and urinary CTX‐I levels in three individuals all showed an association between growth velocity and CTX‐I trajectories, indicating the utility of urinary CTX‐I as a non‐invasive measure of bone growth. In these three bonobos, we were able to avoid an influence of food or daytime on urinary CTX‐I levels, since all samples were collected early in the morning.

## 5 CONCLUSION

Our findings validate urinary CTX‐I as a biologically meaningful indicator of bone metabolism in bonobos. We investigated whether bonobo CTX‐I levels mirrored human CTX‐I patterns in a day‐to‐day comparison, throughout the day, and during expected periods of bone growth. We found a 25% day‐to‐day variability and diurnal decline in urinary CTX‐I levels, consistent with human findings. Age‐related changes in urinary CTX‐I levels demonstrated a decline with increasing age in male and female bonobos; a pattern also found in humans. Interestingly, females exhibited the highest CTX‐I levels at around 4 years of age and not during the expected adolescence period, indicating sex‐specific variations in skeletal growth dynamics. Females also displayed a positive correlation between urinary CTX‐I levels and forearm growth velocity, but males did not. The observed variability in urinary CTX‐I levels underscores the necessity of controlling factors such as diet and activity in future studies. This study establishes the groundwork for utilizing urinary CTX‐I as a valuable tool to assess bone metabolism and growth dynamics in both zoo‐housed and wild bonobos. There are species‐specific assays (e.g., human, mice, and rat) for CTX‐I measurement underscoring the necessity of validating assays for the species under study. However, since Type‐I collagen is the most abundant protein in all vertebrates and has a phylogenetically well‐conserved primary sequence (Makareeva & Leikin, 2014; Pachence et al., 2007), we are confident that measuring urinary CTX‐I levels can be used as a tool for monitoring bone physiology in a wider range of mammals. Therefore, future studies investigating urinary CTX‐I levels have the potential to examine bone metabolism and growth dynamics also in other mammals, thereby advancing our understanding of skeletal physiology across species.

## AUTHOR CONTRIBUTIONS

**Verena Behringer:** Conceptualization (equal); data curation (equal); funding acquisition (supporting); investigation (lead); methodology (lead); project administration (equal); validation (lead); writing – original draft (equal); writing – review and editing (equal). **Ruth Sonnweber:** Formal analysis (lead); investigation (equal); software (lead); visualization (lead); writing – original draft (equal); writing – review and editing (equal). **Gottfried Hohmann:** Conceptualization (equal); project administration (equal); resources (equal); writing – review and editing (equal). **Jeroen M. G. Stevens:** Conceptualization (equal); resources (equal); writing – review and editing (equal). **Jonas Verspeek:** Resources (equal); writing – review and editing (equal). **Tracy L. Kivell:** Conceptualization (equal); funding acquisition (lead); investigation (equal); writing – review and editing (equal).

## FUNDING INFORMATION

This study was funded by the Max Planck Institute of Evolutionary Anthropology and the Deutsche Forschungsgemeinschaft DFG BE 5511/4–1. Further support was provided by the German Primate Center.

## CONFLICT OF INTEREST STATEMENT

We declare we have no competing interests.

## Supporting information


Data S1.

## Data Availability

Source data and R‐code are permanently stored at GRO.data: “Replication Data for: Validating urinary CTX‐I in bonobos,” https://doi.org/10.25625/MVIFWN
