# Supplementary material for: A non‐invasive measure of bone growth in mammals: Validating urinary CTX‐I as a bone resorption marker through long‐bone growth velocity in bonobos
Source: Ecol Evol. 2024 Sep 23;14(9):e70326. doi: 10.1002/ece3.70326 (PMC11417956; doi:10.1002/ece3.70326)
Supplement: Supplementary file 1 — Data S1. [file ECE3-14-e70326-s001.pdf]

Electronic supplementary material

1. *Assay description*

10 µl of standards, controls and (diluted) samples were pipetted into streptavidin -coated microtiter wells. Subsequently, 150 µl of an antibody solution (mixture of biotinylated antibody and peroxidase-conjugated antibody) was added to each well, and the plate, sealed with tape, was incubated for 120 minutes at room temperature in the dark on a shaker at 300 rpm. After incubation, the wells were emptied and washed five times with 300 µl of washing buffer. Then, 100 µl of a chromogenic substrate was added to each well, sealed, and incubated at room temperature, in the dark, on a shaker for 15 minutes. Finally, 100 µl stop solution (sulfuric acid) was added to each well to stop the color reaction. Absorbance was measured at 450 nm with 650 nm as a reference on a plate reader.

2. *Testing for distribution specification in velocity and CTX models*

Prior to model fitting, we assessed the distributions of both log-transformed forearm growth velocity and log-transformed urinary CTX-I levels by plotting histograms. These plots revealed that both variables exhibited a left-skewed distribution. Therefore, we refrained from specifying a gaussian distribution in the respective brms models, but aimed at accounting for the asymmetry in the response variables. To that end, we ran two velocity- and two CTX-I models: the velocity- and the CTX-I models were fit (i) with a skew-normal distribution (which accounts for skewed data with mu (mean or location), sigma (standard deviation or scale parameter), and alpha (skewness parameter) as identity functions), and (ii) then with a Student distribution (which performs well when dealing with a minor skew and outliers).

Both velocity models had the log-transformed velocity values as a response variable, the interaction of a smooth function for age (basis splines with eight knots) with sex as predictors, and a random effects structure (random intercept for the individual with a random slope for the smooth function for age; and a random intercept for zoo). Age and sex were both mean centered and standardized to two standard deviations. The models ran five Markov chains in parallel. Each chain underwent 10,000 iterations without thinning, with the initial 2,000 iterations in each chain for warm-up. Two CPU cores were used. We set the adapt_delta parameter to 0.99.

The CTX-I models were specified exactly as the velocity models, but in addition the time of sample collection was a predictor of log-transformed urinary CTX-I levels.

Posterior predictive checks and posterior distribution plots (Figure S1) revealed that both, the growth velocity as well as the CTX-I model performed better with the skew-normal distribution.

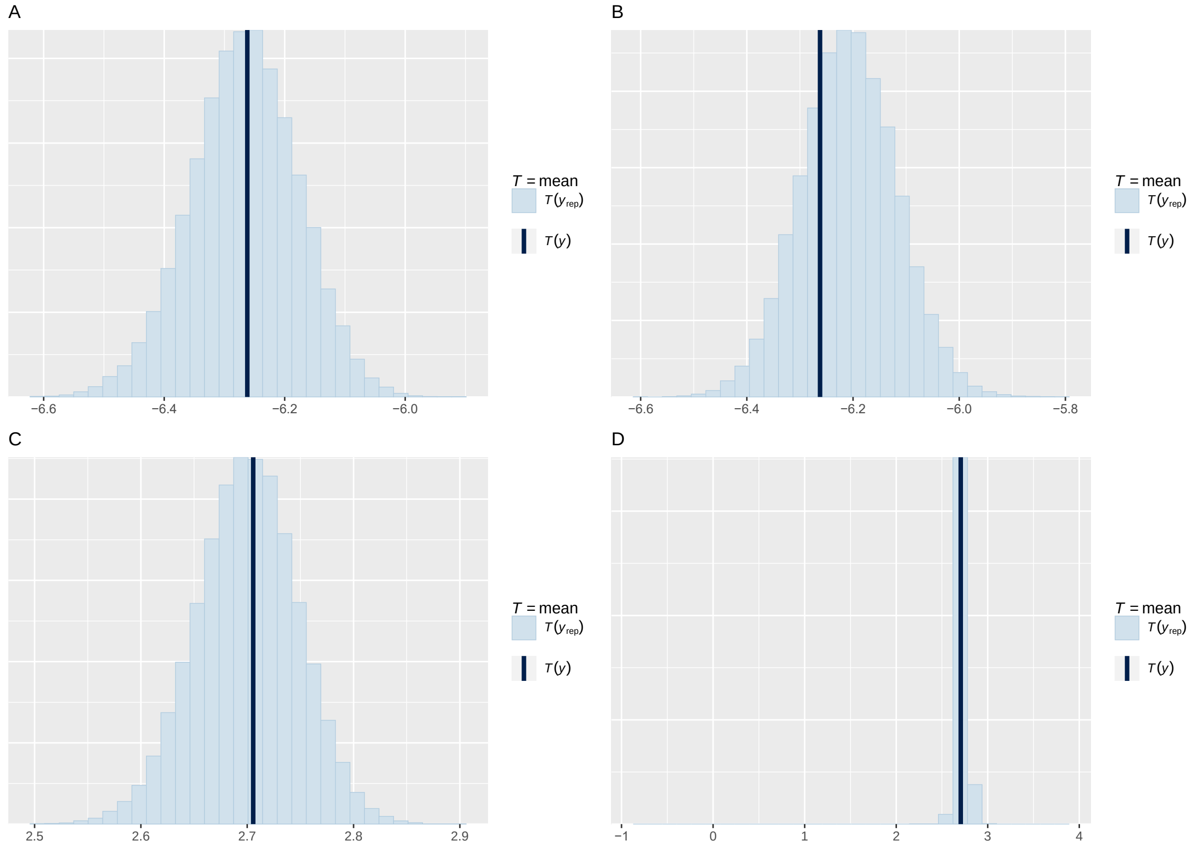

Figure S1: Posterior distribution plots of the growth velocity model specified with a skew-normal distribution (A), the growth velocity model specified with a Student distribution (B), the CTX-I model specified with a skew-normal distribution (C), and the CTX-I model specified with a Student distribution (D). Both the growth velocity as well as the CTX-I model performed better with the skew-normal distribution.

3. *Selection on smooth curve function of age*

We examined different smooth functions of age (with six, eight, and ten knots) in the velocity model. Expected log predictive density values (using loo compare function of the loo package) provide a robust way to assess the out-of-sample predictive performance of Bayesian models (Vehtari et al., 2017). Therefore, we performed model comparison using *loo* for the three different velocity models: (i) one, where the smooth function of age was specified with *six* knots, (ii) one, where the smooth function of age was specified with *eight* knots, and (iii) one, where the smooth function of age was specified with *ten* knots. The three models were compared based on their estimates of predictive performance (Table S1), which showed that the velocity model

with eight knots in the smooth function of age had the highest estimated log predictive density (elpd) among the three models, thus, serving as the reference model. The velocity model containing the smooth function of age with ten knots and the model containing the smooth function of age with six knots exhibited lower elpd values. The differences in elpd values between the models are relatively small and the standard errors associated with these differences are relatively large. Nevertheless, the models generally performed similarly in terms of out-of-sample predictive performance. Based on the loo comparison, we found that all models provide reasonably comparable predictions, with the model containing a smooth function of age with eight knots having a slight edge in terms of estimated predictive performance. We then calculated Bayes R-squared values for each of these models (Table S1) to further evaluate their goodness of fit and the proportion of variance explained. This revealed that the growth velocity model containing the smooth function of age with ten knots had the highest R-square value, indicating that it explains the highest proportion of variance among the models. Finally, visually inspecting posterior predictive checks and posterior distributions suggested that, while all competing models performed well, the growth velocity model containing the smooth function of age with ten knots had the best fit. Taken all this information together, we decided to proceed with the model containing the smooth function of age with ten knots for our further analyses.

Table S1: Comparison of three velocity models, differing in the specification of the smooth function of age. Elpd-difference represents the difference in expected log pointwise predictive density between the respective model and the best-performing model, while SE-difference indicates the standard error of this difference. The R-squared values represent the proportion of variance in the response variable (log-transformed velocity) that is explained by each respective model. A higher Bayes R2 indicates a better fit and a higher proportion of explained variance.

**Velocity model**	**Elpd-difference (SE-difference)**	**R-squared (95% CI)**
Smooth function of age with six knots	-2.6 (2.0)	0.303 (0.242 - 0.361)
Smooth function of age with eight knots	0.0 (0.0)	0.329 (0.271-0.382)
Smooth function of age with ten knots	-0.2 (2.7)	0.350 (0.294 - 0.402)

4. Model checks, prior sensitivity and cross validation results

As different prior choices can influence the posterior estimates and affect the robustness of our findings, we investigated the impact of various prior specifications on the velocity model's performance and results. Using a leave-one-out cross-validation, we compared seven different velocity models (no prior specification, three priors specifying sex-differences in growth velocity across age varying in prior strength, and four priors specifying sex-differences in growth velocity across age). This revealed that all models generalize similarly well to unseen data. Also, expected log predictive density values showed that the models had largely comparable predictive performance. Overall, the results were not sensitive to different prior specifications (Table S2). Similarly, the CTX-model without priors and the CTX-model with priors showed comparable predictive performance in terms of expected log predictive density values and generalization to unseen data.

The growth velocity models shown in Table S2 were used to examine the sensitivity of the results to prior specifications and to conduct a cross validation. All growth velocity models followed the previously described structure (with the smooth function of age specified with ten knots) and differed solely in regards to prior specifications. Priors were set to inform the model about expected sex-differences in arm growth velocity based on.

Table S2: Overview of models used for prior sensitivity analyses and cross validation. Priors were set at specific age splines of the age curve to inform the model about expected sex differences.

model	Prior 1	Prior 2	Prior 3	Prior 4	Prior strength
vel_model20.1_9	No priors				
vel_model20.1_9.1a	0 - 2.5 (spline 1)	4.5 - 7 (spline 3)	7 – 9 (spline 4)	9 – 11 (spline 5)	strong
vel_model20.1_9.1	0 - 2.5 (spline 1)	4.5 - 7 (spline 3)	7 – 9 (spline 4)	9 – 11 (spline 5)	very strong
vel_model20.1_9.2	0 - 2.5 (spline 1)	4.5 - 7 (spline 3)	7 – 9 (spline 4)		strong
**vel_model20.1_9.3**	**4.5 - 7 (spline 3)**	**7 – 9 (spline 4)**	**9 – 11 (spline 5)**		**strong**
vel_model20.1_9.3_weak	4.5 - 7 (spline 3)	7 – 9 (spline 4)	9 – 11 (spline 5)		weak
vel_model20.1_9.3_medium	4.5 - 7 (spline 3)	7 – 9 (spline 4)	9 – 11 (spline 5)		medium

Expected log predictive density (elpd) values showed that the models had largely comparable predictive performance (Table S3).

Table S3: Overview of relative expected log predictive density values (elpd-difference) and associated standard error differences for seven velocity models differing in prior specifications. Lower elpd values indicate better predictive performance.

model	elpd- difference	SE-difference
vel_model20.1_9.1 (reference model)	0.0	0.0
vel_model20.1_9.3_weak	-2.7	1.8
vel_model20.1_9	-3.2	1.7
vel_model20.1_9.2	-3.6	2.1
vel_model20.1_9.3_medium	-3.7	2.3
**vel_model20.1_9.3**	**-5.2**	**2.7**
vel_model20.1_9.1a	-10.3	3.6

The results of the leave-one-out cross validation are shown in Table S4. Here we assess predictive performance by estimating how well the model generalizes to new/unseen data by repeatedly fitting the model to subsets of the data and testing it on the remaining data. This helps to detect overfitting and to get a robust estimate of predictive performance. The data were split into five folds (equal sized subsets of data) for cross-validation.

Table S4: Results of the leave-one-out cross validation. Each row contains elpd values for one model, each column represents one fold of the analysis. The last column shows the average elpd value across all folds. All models perform very similarly, suggesting that the predictive performance is not heavily influenced by prior specifications.

Model	Fold 1 elpd_loo	Fold 2 elpd_loo	Fold 3 elpd_loo	Fold 4 elpd_loo	Fold 5 elpd_loo	Average elpd_loo
**vel_model20.1_9.3**	**-115.5515**	**-119.4442**	**-108.2492**	**-113.8374**	**-121.5798**	**-115.9324**
vel_model20.1_9.3_medium	-115.2037	-119.5187	-107.7959	-113.6964	-120.9696	-115.0369
vel_model20.1_9.3_weak	-115.0189	-119.6759	-107.2034	-113.7254	-120.5333	-115.0314
vel_model20.1_9	-114.8545	-120.5202	-106.7919	-114.3093	-120.1783	-115.1308
vel_model20.1_9.1	-115.279	-120.1312	-105.9344	-112.8944	-119.2272	-114.8934
vel_model20.1_9.1a	-116.3716	-120.6726	-109.5158	-113.8365	-123.4105	-116.7614
vel_model20.1_9.2	-114.9134	-119.8234	-107.7213	-113.4583	-121.179	-115.0195

Furthermore, we tested the CTX-I model with the priors used in the final growth velocity model (but specified to be less informative) and no priors specified. The two models did not differ dramatically in terms of elpd (elpd-difference = -2.3, SE-difference = 2.3 with the model without priors being the reference model) or predictive accuracy results from the leave-one-out cross validation (Table S5).

Table S5: CTX-I model with prior specification and without prior specification.

Model	Fold 1 elpd_loo	Fold 2 elpd_loo	Fold 3 elpd_loo	Fold 4 elpd_loo	Fold 5 elpd_loo	Average elpd_loo
**ctx_model20.1_9.3**	**-25.61688**	**-32.76159**	**-34.33408**	**-35.57964**	**-31.31926**	**-31.92229**
ctx_model20.1_9	-25.98606	-30.78114	-35.2685	-34.61517	-30.61988	-31.45415

References

Vehtari, A., Gelman, A., & Gabry, J. (2017). Practical Bayesian model evaluation using leave-one-out cross-validation and WAIC. *Statistics and Computing*, *27*(5), 1413–1432. https://doi.org/10.1007/s11222-016-9696-4
